# OR14-002 - ANTI IL-1 therapies and pregnancy outcome

**DOI:** 10.1186/1546-0096-11-S1-A269

**Published:** 2013-11-08

**Authors:** HJ Lachmann, H Ozdogan, A Simon, N Stewart, DM Rowczenio, S Ugurlu, T Lane, PN Hawkins

**Affiliations:** 1 National Amyloidosis Centre, University College London Medical School, London, UK; 2 Department of Rheumatology, Cerrahpasa Medical School, Istanbul, Turkey; 3 General Internal Medicine, Radboud University , Nijmegen, Netherlands

## Introduction

Young women with autoinflammatory diseases on long term IL-1 blockade are increasingly asking about the feasibility, safety and outcomes of pregnancy but few data are available. The FDA classes anakinra as pregnancy risk grade B and rilonacept and canakinumab as grade C. The manufacturers advise that there are no data on the outcome of pregnancy or on excretion into breast milk and that anakinra and canakinumab should only be taken if the benefits outweigh risk; for rilonacept the advice is to avoid in pregnancy. The literature contains only 3 reported cases of use of anakinra in pregnancy – all in adult onset Stills disease (AOSD) and with successful outcomes.

## Objectives

To assess pregnancy outcomes in women who had received anti IL-1 therapies in pregnancy.

## Methods

We indentified women who have been exposed to anti IL-1 agents in completed or planned to complete pregnancies under our care. Data were collected on medication, pregnancy outcome, breast feeding and development.

## Results

7 cases were identified; 5 completed, 1 first and 1 second trimester pregnancies. The underlying diseases were: 3 CAPS, 1 TRAPS, 1 FMF, 1 idiopathic pericarditis and 1 AOSD. 3 completed pregnancies (CAPS, TRAPS, pericarditis) were on anakinra from preconception throughout the pregnancy, the 2 current pregnancies (CAPS) were on canakinumab pre conception, 1 switched to anakinra 8 weeks pre conception, the other stopped canakinumab 8 weeks after conception and currently (12 weeks later) is on no treatment. The patient with FMF also had multiple sclerosis with presumed prolonged febrile myalgia refractory to colchicine for which she received 12 weeks of anakinra from 22 weeks. The AOSD patient received anakinra and prednisolone from 22 to 33 weeks. Median maternal age was 30 years (25-38), all were first pregnancies. The 5 completed pregnancies resulted in 5 healthy boys; median gestation 38 weeks (35 to 41). One baby was delivered by caesarean section at 36 weeks for vaginal bleeding (FMF), the others were vaginal deliveries (2 induced); median birth weight 2.59 kg (2.02-3.94), 1 minute APGAR score was 8 in 1 case and 9 in the rest. All were normal on neonatal checks, one had evidence of unilateral reduced hearing at 6 weeks. One was breast fed, the others bottle fed. Follow up data is available on 3 beyond 6 months and their development remains normal.

## Conclusion

These 5 successful pregnancies more than double the number of known outcomes in anakinra treated mothers and provide reassurance to physicians caring for young women. Nonetheless the numbers remain very small and each pregnancy should be assessed and the risks and benefits of continued therapy individually discussed with the potential parents.

## Disclosure of interest

None declared

